# Assessment of Mineral and Phenolic Profiles and Their Association with the Antioxidant, Cytotoxic Effect, and Antimicrobial Potential of *Lycium chinense* Miller

**DOI:** 10.3390/plants9081023

**Published:** 2020-08-13

**Authors:** Muthu Thiruvengadam, Bimal Kumar Ghimire, Seung-Hyun Kim, Chang Yeon Yu, Deog-Hwan Oh, Ramachandran Chelliah, Chang Kwon, Yun-Ju Kim, Ill Min Chung

**Affiliations:** 1 Department of Crop Science, College of Sanghuh Life Science, Konkuk University, Seoul 05029, Korea; thiruv30@gmail.com (M.T.); bimal_g12@yahoo.com (B.K.G.); kshkim@konkuk.ac.kr (S.-H.K.); bks12381@gmail.com (C.K.); suluvisu@gmail.com (Y.-J.K.); 2 Bioherb Research Institute, Kangwon National University, Chuncheon 24341, Korea; cyyu@kangwon.ac.kr; 3 Food Science and Biotechnology, Kangwon National University, Chuncheon 24341, Korea; deoghwa@kangwon.ac.kr (D.-H.O.); ramachandran865@gmail.com (R.C.)

**Keywords:** *Lycium chinense*, MTT assay, MIC assay, microorganisms, secondary metabolites, leaf extract

## Abstract

This study aimed at investigating the *Lycium chinense* Miller leaf extract mineral and phenolic compound profiles as well as antioxidant and antimicrobial potential. We determined the leaf extract mineral composition, identified its major mineral components, and quantified secondary metabolites. We also measured the leaf extract antioxidant potential and found that it varies in a concentration-dependent manner. We observed a significant and higher positive correlation between DPPH and ABTS assays compared with the total phenolic and flavonoid content. Furthermore, our assay results positively correlated with several observed acids, indicating their strong association with the *L. chinense* antioxidant potential. Our cytotoxic assay revealed weak toxicity at higher tested concentrations. Our MIC assay showed that the 80% methanol extract effectively inhibited the growth of *Escherichia coli* Castellani and Chalmers (ATCC35150). The 625-ppm leaf extract completely suppressed the growth of *Staphylococcus aureus* Rosenbach (ATCC13150), *Bacillus cereus* (ATCC 14579), and *Helicobacter pylori* (ATCC43504). These results allow us to understand the indigenous medicinal value of *L. chinense*. Our study suggests that the *L. chinense* leaf extract phenolic compounds possess a good antioxidant activity against free radicals and are effective antimicrobial agents. Finally, the presence and high level of diverse minerals suggest the potential of *L. chinense* for nutraceutical and functional food applications.

## 1. Introduction

Recently, several studies focused on plant phenolic compounds owing to their significant potential for promoting human health and lowering the hazards of various diseases [1,2,3]. Mineral elements and phenolic compounds are considered to play important roles in the antioxidant properties of plants [4,5]. Minerals such as Zn, Cu, and Re reportedly play important roles in the antioxidant potential of plants by serving as cofactors for antioxidant enzymes and cytochromes [6,7]. Other microelements such as K, Na, Mg, Ca, P, S, Co, and Se are important building blocks for several enzymes, including superoxide dismutase (SOD), peroxidase (POX), and catalase (CAT) that protect the cell membrane [6]. Mn is an antioxidant constituent that plays an important role in reactive oxygen species (ROS) scavenging in cells [8,9]. Further, certain trace elements such as Cd and Pb are toxic. Pb accumulation in plants is carcinogenic and causes neurological disorders, whereas Al accumulation in plants is associated with Alzheimer’s disease [10]. Some of these elements, such as Mn, Cu, and Fe, need to be present in balanced proportions, as they are toxic in nature at higher concentrations [11,12]. The daily intake of these elements is important to maintain an antioxidant enzyme balance [13]. However, to date, no evidence is available about the phenolic compound and mineral element composition of plants. Therefore, studying these profiles in plants is essential.

Free radicals are produced during metabolic processes and present serious health risks. ROS, such as O^2−^, OH^−^, and NO, contribute to the degradation of cellular components such as DNA, proteins, and lipids, causing several diseases, including cardiovascular and inflammatory diseases, aging, carcinogenesis, and sclerosis [14]. Therefore, the application of synthetic chemical compounds with higher antioxidant potentials could be useful in treating such diseases [15]. However, such chemical compounds as an antioxidant might cause various human health problems [16].

Synthetic antimicrobial agents are used to prevent microorganism-induced food spoilage [17,18,19]. Moreover, pharmacological studies discovered several new antibiotics for the treatment of various diseases caused by infectious microorganisms. The widespread use of antibiotics is an important factor in the development of multidrug-resistant microbial strains [3,20]. Current research interests are focused on plant-based solutions to develop remedies for such infectious diseases and overcome the aforementioned drawbacks. Furthermore, secondary metabolites, such as phenolic compounds, present in medicinal plants have been proven effective against a wide range of pathogenic microbes, making them potential alternative antimicrobial agents [1,2].

*Lycium chinense* Miller, commonly known as boxthorn, belongs to the family *Solanaceae* and is a perennial plant; it is widely distributed in Korea, China, Japan, and several European countries [21]. In traditional medicine, this plant is widely used as a health food supplement [22], improving vision and wellness [23]. Extracts of this plant were shown to possess certain pharmacological properties including antidiabetic, antiaging [21,23], anti-inflammatory [24], hepatoprotective [25,26], and antioxidant properties [27,28,29]. Several studies have attributed these health benefits to various secondary metabolites, including flavonoids and phenolics [29], alkaloids, terpenoids, organic acids, and their derivatives [30]. Increased evidence of the safety and efficacy of the phytochemicals present in *L. chinense* extracts has been accumulated recently. However, limited information is available on its biological properties and secondary compound compositions. Therefore, in this study, we focused on determining the phenolic compound and mineral content of *L. chinense* extracts and investigating its potential antioxidant and antimicrobial effects. In addition, this study was also extended to elucidate the possible cytotoxic and irritation potential of *L. chinense* leaves. We hypothesized that the phenolic compounds present in the leaf extracts of *L. chinense* contribute to its antioxidant activity and the inhibition of microbial growth.

## 2. Results and Discussion

### 2.1. Mineral Elements

An imbalance in the intake of mineral elements could lead to various health problems [10]. Moreover, mineral elements play important roles in the antioxidant properties of plants [31]. Therefore, considering the importance of trace elements, it is important to analyze the plant mineral element concentration and composition profile. In the present study, we analyzed the mineral element content of the leaf extracts using the ICP-MS method and observed significant differences in the mineral element concentrations (Table 1 and Table 2).

The seven most dominant minerals (in decreasing order) were as follows: Ca (92.77) > Mg (28.68) > Mn (1.696) > Fe (0.875) > Al (0.551) > Zn (0.303) > Ba (0.269). Among the minerals, Cu exhibited the lowest concentration (0.031 ± 0.001 mg/mL). The presence of elements such as As, Cd, Pb, Hg, and Al beyond certain concentrations can be toxic [32]. Interestingly, in the present study, the concentrations of these elements were within the environmental safety limits. The concentrations of other elements such as Zn, Fe, and Cu were observed to be within the permissible levels. It is worth mentioning that the *L. chinense* leaf extracts could serve as good sources of minerals (for dietary intake) and play important roles in maintaining health.

### 2.2. Correlation between Antioxidant Properties and Minerals

Several metal elements are known for their antioxidant properties. In the present study, a moderate and positive correlation was observed between the mineral elements and antioxidant activity, indicating that these elements contributed to the antioxidant properties of the plant (Table 3).

A positive and significantly high correlation (r = 0.997, *p* = 0.05) was observed between Se and the antioxidant activity (ABTS radical scavenging activity). Conversely, a strong and positive correlation was recorded between As, Ba, Ca, Cr, Mg, and Ti and the total phenolic content (TPC). Minerals such as Bi, Cu, Fe, Li, Mn, Ni, Pb, and Se positively correlated with the total flavonoid content (TFC), indicating that these minerals might be a part of the phenolic compounds and play vital roles in the antioxidant (AO) properties of the leaf extracts. In the present study, a strong and positive correlation was observed between minerals such as Bi, Co, Cu, Fe, Cr, and Pb and 1,1-diphenyl–2-picrylhydrazyl (DPPH) and 2,2′-azino-bis-3-ethylbenzthiazoline-6-sulphonic acid (ABTS). The present study also corroborated with the study of Perna et al. [33], describing a significant and positive correlation between the metal content and antioxidant activities. This study also corresponds to the study conducted by Solayman et al. [34], as we report a close and positive correlation between metal ions and the AO properties. Moreover, metals such as Cu, Zn, and Mn are essential co-factors for and integral parts of the superoxide dismutase enzyme required for the conversion of superoxide into hydrogen peroxide [35]. However, as per our study, other mineral elements such as Al, Ca, Li, Mg, Se, Ag, Ti, and Zn might not affect the antioxidant properties as non-significant correlations were observed between them.

### 2.3. The TPC, TFC, and Phenolic Compound Composition of L. chinense Leaf Extracts

The total phenolic and flavonoid content of the *L. chinense* leaf extracts, determined using a calibration curve (R^2^ = 0.988), was 7.55 ± 0.22 gallic acid equivalent/g, and the total flavonoid content (R^2^ = 0.989) was 0.02 ± 0.001 quercetin equivalent/g (Table 4). The determination of phenolic compounds using the Folin-Ciocalteu method tends to be inaccurate owing to the presence of non-phenolic compounds such as sugar and amino acids in the tested samples.

**Figure 1:**
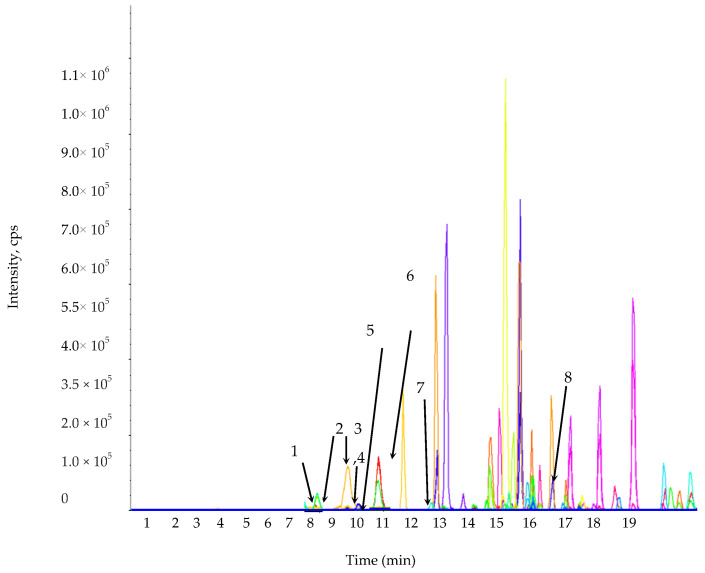
Multiple reaction monitoring mode (MRM) ion chromatogram of the selected phenolic compound standards. 1. Protocatechuic acid; 2. Chlorogenic acid; 3. Rutin; 4. *p*-Hydroxybenzoic acid; 5. Caffeic acid; 6. Gentisic acid; 7. *p*-Coumaric acid; 8. Salicylic acid.

**Figure 2:**
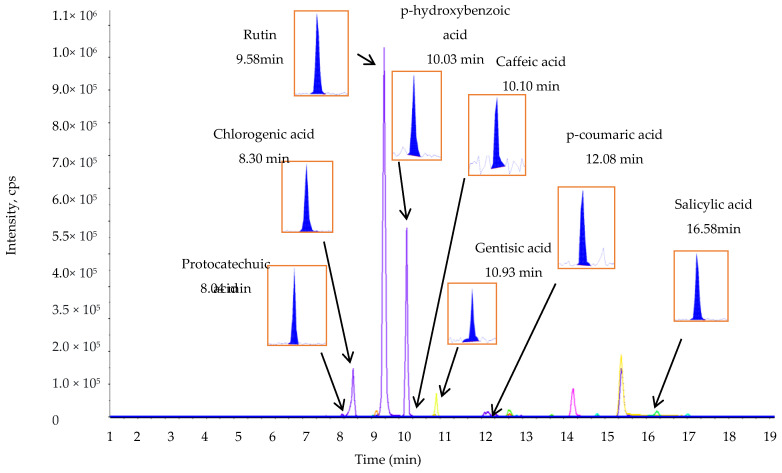
Representative MRM ion chromatogram of the phenolic compounds from the *L. chinense* leaf extracts. The extract ion chromatograms of the individual phenolic metabolites with small peaks are given in the rectangular boxes with their retention time.

The phenolic compounds vary widely in their structure (owing to the presence of a hydroxyl group in their chemical structure) and are responsible for various biological activities [36]. LC-MS/MS is widely used for the identification and quantification of several phenolic compounds [37]. This study identified and quantified phenolic compounds in the *L. chinense* leaf extracts using LC-MS/MS (Figure 1 and Figure 2).

The concentrations of the phenolic compounds present in the sample were determined on the basis of the peak area. Among the quantified compounds, protocatechuic acid (1198.70 ± 5.00 µg g^−1^), hydroxybenzoic acid (529.70 ± 6.15 µg g^−1^), and *p*-coumaric acid (466.90 ± 6.50) were the most predominant phenolic compounds, accounting for more than 60% of the total phenolic compound concentration, whereas caffeic acid was the least abundant phenolic acid (67.60 ± 3.00 µg g^−1^) as shown in Table 5.

### 2.4. Antioxidant Activity

The antioxidant potential of the *L. chinense* leaf extract was determined using the DPPH and ABTS assays (Table 4). The results showed that the leaf extract has a greater ability to scavenge the DPPH and ABTS radicals with IC50 values of 70.99 ± 7.95 and 1772.31 ± 10.87, respectively. Although the antioxidant potential data obtained between the antioxidant assays were somewhat different, they highly correlated between themselves. This difference could be due to the variation in the response of the antioxidant substances to different radicals present in each assay. Meanwhile, higher positive correlations were observed between the TPC, TFC, and antioxidant properties (Table 6).

Several studies have attributed the antioxidant potential of plants species to their phytochemical components, including phenolic compounds, which effectively act as reducing agents, hydrogen donors, and singlet oxygen quenchers [38,39]. In the present study, the Pearson’s correlation coefficients between the phenolic compounds and antioxidant potentials (1/RC50) varied greatly. The DPPH assay exhibited higher positive correlation with the gentisic acid (r^2^ = 0.900, *p* < 0.05), *p*-coumaric acid (r^2^ = 1, *p* < 0.01), and caffeic acid (r^2^ = 0.999, *p* < 0.05), whereas the ABTS radical scavenging activities positively correlated with the *p*-coumaric acid (r^2^ = 0.999, *p* < 0.05), hydroxybenzoic acid (r^2^ = 0.999, *p* < 0.05), gentisic acid (r^2^ = 0.999, *p* < 0.05), and protocatechuic acid (r^2^ = 0.997, *p* < 0.05), indicating that these compounds might be strongly associated with the antioxidant potential of *L. chinense*. Other studies deducted positive associations between the plant phenolic compounds and antioxidant activities [38,40,41], which were also in a good agreement with the present study, indicating the higher contribution of these compounds to the antioxidant properties of the leaf extracts. It has been reported that phenolic compounds, such as phenolic acid and flavonoids, act as antioxidants by chelating metal cations, scavenging radicals, or donating hydrogen ions or electrons [42,43]. It has also been reported that the antioxidant properties of individual phenolic compounds depend on the number of hydroxyl (OH) groups and their arrangement in the aromatic rings as well as the availability of electron-donating ions [44,45,46]. Other studies reported the existence of synergistic or additive effects between the individual phenolic compounds that contribute to plant antioxidant activities [47]. Thus, the results of the present study indicate that the phenolic compounds of the leaf extracts and their synergistic effects are important contributors to the antioxidant properties of *L. chinense*.

### 2.5. Cytotoxic Effect

**Figure 3:**
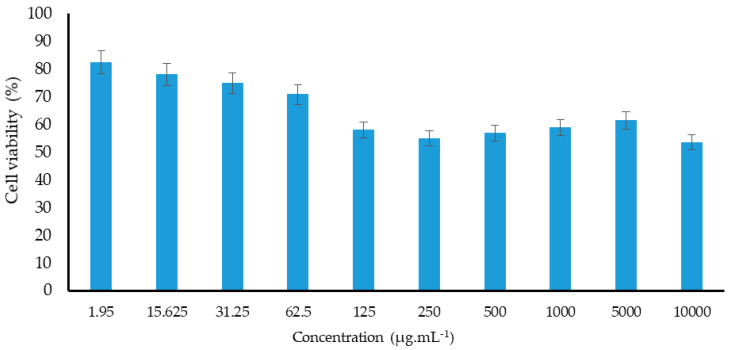
The effect of leaves extracts concentration on cell viability on NIH 3T3 cells lines.

**Figure 4:**
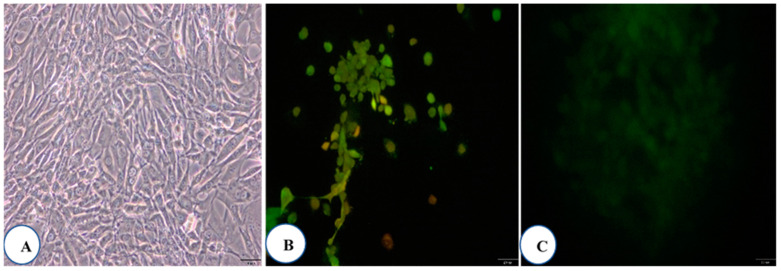
Cytotoxic effect of the leaf extracts (IC_50_ concentrations) on the NIH 3T3 cell lines. (**A**) Bright field, (**B**) AO/EB staining, (**C**) ROS.

The MTT assay has been widely used as a method to assess cell viability through the production of water soluble products with increased sensitivity and higher flexibility in the operation as well as cost and time efficiency [48]. To quantitatively estimate living cells, the amount of formazan crystals produced during metabolic processes in active NIH 3T3 cell lines is determined [49]. *L. chinense* leaf extracts reportedly exhibit higher cytotoxic effects in cancer cells [50]. However, the cytotoxic potential of the concentrated leaf extract of *L. chinense* on normal cells and its half maximal inhibitory concentration (IC50) has not yet been determined. Therefore, in this study, we documented the cytotoxicity of various *L. chinense* leaf extract concentrations in the NIH-3T3 cell line (Table 7). As shown in Figure 3 and Figure 4, the *L. chinense* leaf extracts exhibited cytotoxicity in a concentration-dependent manner (50 mg/mL).

The results showed that higher leaf extract concentrations reduced the NIH-3T3 cell number. The cell viability was not significantly affected when the concentration of the extracts was between 1.95 and 62.5 ppm. However, the cell viability significantly reduced (nearly by 53%) when the leaf extract concentration increased to 10,000 ppm. However, it has been observed that higher than the required amounts of bioactive compounds might cause an imbalance in their function, which might not help prevent cellular oxidative stress and thus lead to cell death [51].

Several studies indicated that phenolic compounds are the main phytochemicals responsible for the antioxidant and antimicrobial properties of plant extracts [52,53,54]. These phytochemicals are also responsible for inducing cytotoxicity. In the present study, the phenolic compound profile of the *L. chinense* leaf extracts was investigated, which exhibited high concentrations of polyphenols, such as protocatechuic acid, hydroxybenzoic acid, *p*-coumaric acid, genitisic acid, salicylic acid, caffeic acid, and rutin, which could be responsible for the cytotoxic properties. Moreover, previous studies indicated that phenolic compounds have implications in strong cytotoxicity. For instance, higher rutin, quercetin, chlorogenic acid, and luteolin concentrations effectively inhibited the growth of NIH-3T3 cells [55,56,57], whereas quercetin derivatives, such as chloronaphthoquinone quercetin, prevented the proliferation of NIH 3T3 cells [58]. Moreover, we described, in this study, a strong positive correlation between cytotoxicity and protocatechuic acid (r = 0.957, *p* < 0.01), chlorogenic acid (r = 0.998, *p* < 0.05), *p*-coumaric acid (r = 0.984, *p* < 0.05), or salicylic acid (r = 0.998, *p* < 0.05), as presented in Table 7, indicating that the higher concentrations of these compounds might be associated with *L. chinense* cytotoxicity. However, further studies are still necessary to identify the phenolic compounds responsible for this effector mechanism.

### 2.6. Irritation Potential of Leaf Extracts Measured by the HET–CAM Assay

**Figure 5:**
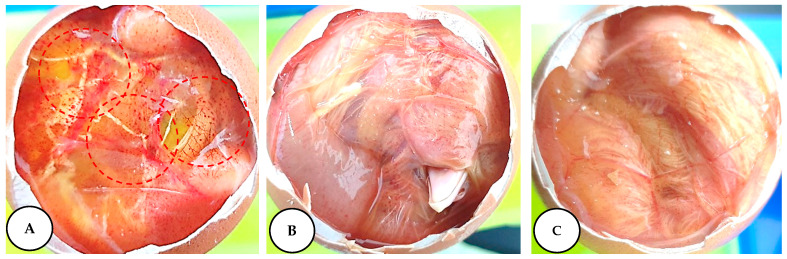
The hen’s egg test on the chorioallantoic membrane (HET–CAM) assay. (**A**) NaOH (0.1 M) positive control; (**B**) negative control (distilled water); (**C**) test sample.

The hen’s egg test on the chorioallantoic membrane (HET–CAM) is considered a rapid and cost effective approach to examine the irritation potential of leaf extracts used in the cosmetic industry and in the development of cosmeceuticals [59]. Different concentrations of leaf extracts and 0.1 M NaOH (positive control) are used to assess the irritation potential of the leaf extracts using different parameters (hemorrhage, coagulation, and lysis), and changes were observed as shown in Figure 5.

We could conclude, from the results of this experiment, that lower extract concentrations did not exhibit embryonic toxicity (Table 8). In the present study, higher extract concentrations showed a certain level of irritation compared to the lower concentrations. The results indicated that lower concentrations of antioxidant compounds present in the leaf extracts are safer for topical application and beneficial for product preparation.

### 2.7. Antimicrobial Activity

#### 2.7.1. Minimum Inhibitory Concentration (MIC)

The antimicrobial activity of the *L. chinense* leaf extracts was tested against several pathogenic Gram-positive and Gram-negative bacterial strains, assessed by determining the inhibition zone and MIC (Table 9). The MIC assay showed that the 80% methanol extracts of *L. chinense* effectively inhibited the growth of the *E. coli strains* (MIC = 125 µg mL^−1^). The leaf extracts completely suppressed the growth of *S. aureus* and *H. pylori* at a concentration of 625 ppm. However, none of the tested microorganisms were susceptible to the leaf extracts. For instance, the *K. pneumonia* and *S. typhimurium* strains were less sensitive to the plant extracts, as indicated by their higher MIC values (>1000).

#### 2.7.2. Disc Diffusion Test

The zone of inhibition of the leaf extracts at a concentration of 1000 µg/mL exhibited a significant difference in the case of all selected bacterial strains (Table 10).

**Figure 6:**
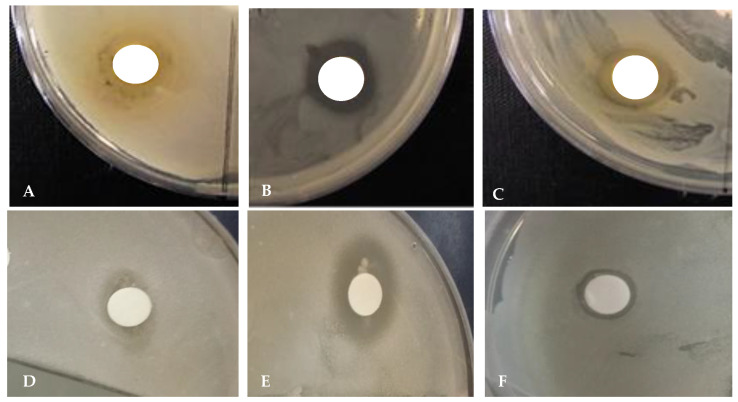
The zone of inhibition of the *L. chinense* methanol extract, (**A**) *E. coli*; (**B**) *B. cereus*; (**C**) *H. pylori;* (**D**) *B. subtilis*; (**E**) *S. aureus*; (**F**) *S. typhimurium*.

Among the tested microbes, *E. coli* was more susceptible; it was effectively inhibited by the leaf extract (zone of inhibition = 1.8 cm). Further, *K. pneumoniae* was the least susceptible (zone of inhibition = 0.5 cm). The extracts showed low to intermediate inhibitory effects on *S. aureus* and *S. typhimurium*, with an inhibition zone of 0.9 cm (Figure 6).

The antimicrobial activity of the *L. chinense* leaf extract might be due to the phytochemicals present in these plants, which are rich in phenolic acids, such as protocatechuic, gentisic, *p*-hydroxybenzoic, chlorogenic, *p*-coumaric, salicylic, and caffeic acid, as well as flavonoids (rutin). Several previous studies observed reductions in microbial populations upon treatment with the phenolic compounds of plant extracts [60,61,62,63]. For instance, *p*-hydrobenzoic acid effectively inhibited the growth of *Staphylococcus aureus* [64]. In another study, chlorogenic acid was found to be the most effective against *E. coli*, *S. aureus,* and *B. subtilis* [65]. These compounds induced an increase in the outer- and plasma-membrane permeability of pathogenic microorganisms, leading to their death [65]. Therefore, it is likely that the higher phenolic compound concentrations of the *L. chinense* leaf extract could be associated with its antimicrobial activity.

## 3. Materials and Methods

### 3.1. Chemicals

All chemicals used in the present study were of analytical grade. Compounds such as the Folin-Ciocalteu reagent, quercetin, kanamycin, vancomycin, α-tocopherol, tert-butyl-4-hydroxy toluene (BHT), 2,2-diphenyl-1-picryl-hydrazyl-hydrate (DPPH), 2,2′-azino-bis-3-ethylbenzthiazoline-6-sulphonic acid, 2,4,6-tri(2-pyridyl)-s-triazine (TPTZ), and gallic acid were obtained from Sigma-Aldrich Chemical Co. (St. Louis, MO, USA). Hydrogen peroxide (H_2_O_2_) was obtained from Sigma-Aldrich (Seoul, Korea), and nitric acid (HNO_3_) was purchased from Showa Chemical Industry Co. Ltd. (Tokyo, Japan). Ultrapure distilled water, which was used in the analysis, was purchased from the Zeneer power 1 system (Human Corporation, Seoul, Korea). The 19 multielement standards used in the analysis (micro- and macro-elements) were purchased from Perkin Elmer (Seoul, Korea). The multielement stock solutions were obtained from Quality Control Standard 26, (Inorganic Ventures, Christiansburg, VA, USA). All analytical equipment required for sample preparation and mineral quantification were cleaned with HNO_3_ (2%, *v*/*v*) and rinsed thrice with ultrapure water.

### 3.2. Sample Digestion

Leaves extracts of *L. chinense* were pre-digested in HNO_3_ as previously described (US Environmental Protection Agency, 2007). Briefly, 0.5 g of the sample was mixed with 7 mL of 70% HNO_3_ at room temperature for 6 h. After incubation, 1 mL of H_2_O_2_ was added to the mixture. The mixture was microwave digested by increasing the temperature to 180 °C for 15 min. The digestion was complete when the sample turned colorless. The final volume of the digested sample was adjusted to 50 mL by adding ultrapure distilled water. Blank samples were prepared via the same method.

### 3.3. Instrumentation and Quantification of Minerals

Inductively coupled plasma atomic emission spectroscopy (ICP-AES) (Optima 7300 DV, Perkin Elmer, Korea) was used to quantify the concentrations of the 19 mineral elements in the leaf extracts of *L. chinense*. The operational conditions and optics used for the determination of the concentrations of the different mineral elements were as follows: 15 L min^−1^ axial mode plasma, 0.2 L min^−1^ auxilliary; 0.65 L min^−1^ nebulizer; 1300 W RF powder; and 1.5 mL min^−1^ flow rate. Except for K and Na, the radial mode of the ICP-AES was used to quantify the mineral elements. In contrast, the axial mode was used for K and Na. Approximately 3–7-point calibration curves were used for quantitation, and the concentration ranges for the 19 elemental STDs are shown in Table 1.

The limit of quantitation (LOQ) and limit of detection (LOD) for the 19 elements were calculated using the following calibration curve:

LOD = 3 × SD/S, LOQ = 10 × SD/S,
(1)

where SD is the standard deviation of a response and S is the slope of the calibration curve.

### 3.4. Preparation of Plant Extracts

*L. chinense* seedlings were grown in an experimental field of Kangwon National University, South Korea (37°52′09.53″ N; 127°44′42.82″ E; at an altitude of 100 m). The leaves of 2-year-old *L. chinense* were collected in early November 2019. The harvested leaf sample was immediately washed with distilled water. Then, the collected leaf samples were sliced and freeze-dried for 24 h. Approximately 2 g of the freeze-dried fine powder sample was mixed with 20 mL of 80% methanol at room temperature (25 °C). Then, the mixture was filtered through a filter paper (Whatman No. 1, Maidstone, UK) to remove the debris. The solvents were evaporated at 40 °C in a rotary evaporator (Eyela, SB-1300, Shanghai Eyela Co. Ltd., China). The residual extracts were dissolved in 80% methanol (300 mL) and stored at 4 °C until further use.

### 3.5. Determination of Total Phenolic Content

The total phenolic content was determined according to the method described by Singleton and Rossi [66]. Initially, in a 10-mL test tube, 100 μL of leaf extract (1 mg mL^−1^) and 50 μL of Folin-Ciocalteu (1 M) were mixed with 1.85 mL of distilled water and incubated at room temperature for 5 min. Then, after 5 min, 400 μL of sodium carbonate (Na_2_CO_3_) was mixed into the solution, followed by the addition of distilled water to obtain a final volume of 4 mL. Deionized distilled water served as a blank. The reaction solution was maintained at room temperature for 1 h in the dark. The absorbance value of the reaction mixture was measured using a spectrophotometer (Jasco V530 UV-VIS spectrophotometer, Tokyo, Japan) at 725 nm against the blank. The TPC was expressed in terms of the gallic acid equivalent (GAE) per g of the dry sample.

### 3.6. Estimation of Total Flavonoid Content

The total flavonoid content (TFC) was determined following the method described by Moreno et al. [67]. In a 10-mL test tube, 500 µL of leaf extract (1 mg mL^−1^) was added to 100 µL of 10% aluminum nitrate and 100 µL of potassium acetate (KCH_3_COO) (1 M). To the reaction mixture, 4.3 mL of 80% ethanol was added to achieve a final volume of 5 mL. After mixing thoroughly, the reaction mixture was incubated at room temperature for 50 min. The absorbance of the mixture was recorded against the blank using a spectrophotometer (Jasco V530 UV-VIS spectrophotometer, Tokyo, Japan) at 415 nm. The total flavonoid content was expressed in terms of the quercetin equivalent (QE) per g of the dry sample.

### 3.7. Estimation of Phenolic Compounds by Liquid Chromatography-Mass Spectrometry/Mass Spectrometry (LC-MS/MS)

The phenolic compound concentration and composition were identified in *L. chinense* using a liquid chromatography-mass spectrometry/mass spectrometry (LC-MS/MS) system, as described previously by Chung et al. [68]. Pumps (Agilent 1200, Agilent Technologies, Palo Alto, CA, USA) and an autosampler (Agilent 1100 series, Agilent Technologies, Palo Alto, CA, USA) coupled to an API 2000 series, mass spectrometer (Applied Biosystems, Ontario, Canada) were integrated to the LC system. The chromatographic separation of phenolic compounds was performed using a C18 column (4.6 × 250 mm, 5 µm). A negative ion mode was used. The following parameters were used to determine the phenolic compounds present in the samples: Nebulizer gas pressure, drying gas pressure, collision gas pressure, and curtain gas pressure, set at 40, 70, 2, and 20 psi, respectively. The drying gas temperature and capillary voltage were set to 350 °C and 4.5 kV, respectively. The mobile phase comprised 0.1% formic acid (HCOOH) (*v*/*v*) in water (mobile phase A) and 0.1% acetonitrile (C2H3N) in water (95:5, *v*/*v*) (mobile phase B). The following mobile phase gradient elution program was used for the efficient separation of compounds: 10–40% B for 0–10 min; 40–50% B for 10–20 min; 50–100% B for 20–25 min; 100–10% B for 25–26 min; and 10% B for 26–30 min. During the experiment, the column temperature was maintained at 25 °C. The volume of the injected sample was 10 µL. The mobile phase was eluted at a constant flow rate of 0.7 mL min^−1^. Electrospray ion source (ESI) was used as the source for recording the mass-spectrometry data; the process was performed in the negative mode under the multiple reaction monitoring (MRM) mode. Different mass spectrometric parameters, such as entrance potential (EP), collision energy (CE), declustering potential (DP), cell entrance potential (CEP), and collision cell exit potential (CXP), were determined for each MRM transition monitored. The analysis of allelochemicals for each sample were performed in triplicate.

### 3.8. Antioxidant Activity

#### 3.8.1. Evaluation of the 1,1-Diphenyl–2-Picrylhydrazyl (DPPH) Radical Scavenging Assay

The free radical scavenging potential of the leaf extract of *AJ* was measured in vitro using the DPPH assay following the method described earlier by Xing et al. [69]. Briefly, 200 µL of sample extract (0.05–10 mg mL^−1^) was added to 4.5 mL of DPPH (0.004% in methanol). The reaction mixture was mixed thoroughly and maintained at 25 °C for 40 min. Then, the absorbance of the reaction mixture was measured using a spectrophotometer (Jasco V530 UV-VIS spectrophotometer, Tokyo, Japan) at 517 nm. A-tocopherol was used as the reference standard antioxidant. The free radical scavenging activity was estimated by the following equation:

DPPH scavenging activity = (Abs_control_ − Abs_sample_)/Abs_control_ (2)

where Abs_control_ is the absorbance of the reaction mixture without the leaf extract. Abs_sample_ is the absorbance of the reaction mixture with the leaf extract. The IC50 value was determined from the plotted graph. Calculated inhibitory concentration (IC_50_) values indicate the amount of test sample needed to inhibit or scavenge 50% of the radicals present in the reaction mixture.

#### 3.8.2. Evaluation of the 2,2′-Azino-bis-3-Ethylbenzthiazoline-6-Sulphonic Acid (ABTS) Assay

The 2,2′-azino-bis-3-ethylbenzthiazoline-6-sulphonic acid (ABTS), also known as the ABTS cation scavenging assay was carried out following the method described previously [70]. Briefly, the ABTS solution was prepared by reacting 7.4 mM ABTS and 2.6 mM potassium persulphate in an equal ratio (1:1, *v*/*v*). Then, the reaction mixture was maintained at room temperature for 12 h. The reaction mixture was then diluted with 80% methanol, and the absorbance of the reaction mixture solution was observed at 734 nm using a spectrophotometer (Jasco V530 UV-VIS spectrophotometer, Tokyo, Japan). For the spectrophotometric measurement of the samples, 1 mL of diluted ABTS and 100 mL of the sample were mixed. Trolox at various concentrations (500–1000 µM) was used to make the standard curve. The ability to scavenge ABTS radicals of the sample was calculated by the following equation:

ABTS scavenging activity = (Abs_control_ − Abs_sample_)/Abs_control_ × 100 (3)

where Abs_control_ is the absorbance value of the ABTS solution without the test sample. Abs_sample_ is the absorbance value of the ABTS solution with the test sample.

### 3.9. Cytotoxicity

The cell line NIH/3T3 ATCC^®^ CRL-1658™ (Embryonic fibroblast) was provided by the School of Biomedical Science, Kangwon National University, South Korea. Each cell line was cultured in a suitable medium to obtain the desired growth, and the growth curve of the cell line was plotted. The cytotoxicity of the leaf extracts was evaluated by treating the NIH/3T3 cell line with the 3-(4,5-dimethyl thiazol-2-yl)-2,5-diphenyl tetrazolium bromide solution (MTT assay) according to a method described elsewhere [71]. The NIH/3T3 cells (at a density of 6 × 10^3^ per well 100 mL of the medium) were grown in a 96-well microtitration plate. After 24 h of incubation, the cells were treated with different concentrations (the maximum concentration was 50 μg·mL^−1^) of the leaf extracts for 72 h. The MTT solution (5 mg·mL^−1^ of final concentration) was added to each well and incubated at 37 °C for 4 h. Then, the supernatant was removed, and the resultant formazan crystals were dissolved in dimethyl sulfoxide (DMSO) (Sigma, MO, USA). The amount of produced formazan was directly proportional to the number of living cells. The absorbance was measured using a microplate reader (Thermofisher Scientific Instrument Co. Ltd., Shanghai, China) at 570 nm. The MTT assay for the cytotoxicity of commercially available identified compounds was performed similarly as described above for leaf extracts. Tamoxifen (Sigma, MO, USA) was used as the positive control in the present study.

% cell viability = A570 of treated cells/A570 of control cells × 100
(4)

### 3.10. Assessment of Irritation Potential of L. chinense Leaf Extracts in a Fertile Chicken Egg

The antiallergic properties of leaf extracts was determined by the HET-CAM test (Hen’s egg chorioallantoic membrane test). Fresh and fertilized eggs where purchased from a chicken farm. The fertilized eggs were incubated at 37 °C with a relative humidity of 60%. The eggs were rotated manually each day by 180° to ensure the proper development of the embryo. On the ninth day, the incubated eggshell was scratched around the air sack and opened up. Carefully, the cell membranes were removed to expose the CAM. The leaf extracts (dissolved in DMSO) at a volume of 0.2 mL were treated to the CAM. The irritant effect of leaf extracts on blood vessels, capillaries, and albumin was examined and scored (between 0 and 21) for each egg on the basis of hemorrhage, coagulation, and lysis for 300 s. The ocular irritation index (OII) was then obtained by the following equation:

OH = (301 − h) × 5/300 + (301 − 1) × 7/300 + (301 − c) × 9/300
(5)

where h is the time to beginning of hemorrhage, l time to lysis, and c time to coagulation. The following classification was used: OII ≤ 0.9, slightly irritating; 0.9 < OII ≤ 4.9, moderately irritating; 4.9 < OII ≤ 8.9, irritating; and 8.9 < OII ≤ 21, severely irritating.

### 3.11. In Vitro Assay for Antimicrobial Activity

#### 3.11.1. Disc diffusion Method

Selected pathogenic bacterial strains were acquired from the Department of Food Science and Biotechnology, Kangwon National University, South Korea. Leaf extracts were tested against *Bacillus cereus* (ATCC 14579), *Klebsiella pneumonia* subsp. pneumoniae (Schroeter) Trevisan ATCC 9621, *Staphylococcus aureus* Rosenbach (ATCC13150), *Salmonella enteritidis typhimurium* Kauffmann and Edwards (ATCC14028), *Bacillus subtilis* (KCCM 11316), *Escherichia coli* Castellani and Chalmers (ATCC35150), and *Helicobacter pylori* (ATCC43504). The antimicrobial activity of the leaf extract of *L. chinense* was evaluated by performing the agar disc diffusion method described elsewhere [72]. Initially, bacterial strains were spread on the solid MRS agar medium at room temperature. Leaf extracts (50 µL each) at a concentration of 1 mg/mL of the dried plant sample were used to soak filter paper discs (6 mm, Whatman, no. 3). The soaked filter paper discs were maintained at room temperature for 10 min to evaporate the methanol and subsequently placed on the MRS medium containing the tested microbial strains. Kanamycin or tetracycline was used as the positive control. Then, the culture plates were incubated at 37 °C for 18–24 h.

#### 3.11.2. Minimum Inhibitory Concentration (MIC)

The minimum inhibitory concentration (MIC) required to inhibit the growth of microorganisms was carried out according to the method described by Kobayashi et al. [73]. The leaf extract was dissolved in 80% methanol to obtain a concentration of 10 mg mL^−1^. Then, 20 µL of the 10 mg mL^−1^ plant extract solution and 180 µL of standard-grown bacterial culture were dispensed to the first well of 96-well assay microplates (SPL, Life Science Co. Ltd., Pocheon, South Korea). The leaves extracts were further diluted by a two-fold dilution series to give a final concentration of 7.8 µg mL^−1^, and the final volume in each well was 200 µL. The tested bacterial strains were incubated at 37 °C for 24 h. The amount of plant sample (lowest concentration) that produce no visible growth of bacteria in the first 24 h of incubation compared with the control was considered the MIC. Tetracycline was used as the positive control.

### 3.12. Statistical Analysis

Statistical analysis was recorded as the mean ± standard deviation using the analysis of variance (ANOVA). Significant differences between the parameters were determined by Duncan’s multiple comparison test at *p* < 0.05 and *p* < 0.01. The interrelationship between minerals and phenolic compounds with antioxidant and cytotoxic properties were assessed by Pearson’s correlation coefficient using SPSS version 20 (SPSS, 2011). All experiments were performed in triplicate.

## Figures and Tables

**Table 1:** Calibration curves, limit of detection (LOD), and limit of quantitation (LOQ) of the 19 elements examined in this study.

Element	Concentration ^a^ (mg L^−1^)	Linearity (r^2^)	Slope (S)	Response SD	RSD ^c^ (%)	LOD ^b^ LOQ ^b^	
						µg mL^−1^	
Al	0.5–10	0.999932	3730	358.88	0.96	0.288643	0.962145
As	0.5–10	0.999876	47.10	3.54	0.75	0.225478	0.751592
Ba	0.5–10	0.999825	5071	505.09	0.99	0.298811	0.996036
Bi	0.5–10	0.999907	286.5	38.39	1.34	0.401990	1.339965
Ca	0.5–10	0.999576	8157	1103.17	1.15	0.405726	1.352421
Cd	0.5–10	0.999812	2007	156.53	0.78	0.233976	0.779920
Co	0.5–10	0.999131	1197	111.51	0.93	0.279474	0.931579
Cr	0.5–10	0.999841	4921	522.49	1.06	0.318527	1.061756
Cu	0.5–10	0.999852	19,530	1778.00	0.91	0.273118	0.910394
Fe	0.5–10	0.999803	4031	352.03	0.87	0.261992	0.873307
Li	0.5–10	0.999783	32,120	3995.40	1.24	0.373169	1.243898
Mg	0.5–10	0.999738	19,920	2106.85	0.94	0.317297	1.057656
Mn	0.5–10	0.999901	31,120	3071.07	0.98	0.296054	0.986848
Ni	0.5–10	0.999757	1951	174.43	0.89	0.268216	0.894054
Pb	0.5–10	0.999792	277.0	37.92	1.37	0.410686	1.368953
Se	0.5–10	0.999913	72.12	8.72	1.21	0.362729	1.209096
Ag	0.5–10	0.999942	20,240	1753.28	0.86	0.259874	0.866245
Ti	0.5–10	0.999927	38,130	2907.65	0.76	0.228769	0.762562
Zn	0.5–10	0.999799	5112	378.98	0.74	0.222406	0.741354

^a^ The calibration curve was obtained using 3–7 different concentrations of a standard solution for each element. ^b^ The LOD and LOQ were determined using each calibration curve as follows: LOD = 3 × SD/S and LOQ = 10 × SD/S, where SD is the standard deviation of the response and S is the slope of each calibration curve.

**Table 2:** Mineral content in the *Lycium chinense* leaf extracts.

Element	Concentration (µg mL^−1^)
Al	0.389 ± 0.0053
As	0.046 ± 0.0319
Ba	0.048 ± 0.0023
Bi	0.062 ± 0.0197
Ca	69.17 ± 0.5610
Cd	0.088 ± 0.0004
Co	0.133 ± 0.0025
Cr	0.094 ± 0.0012
Cu	0.016 ± 0.0017
Fe	0.028 ± 0.0028
Li	0.038 ± 0.0010
Mg	15.40 ± 0.1040
Mn	0.345 ± 0.0025
Ni	0.110 ± 0.0019
Pb	0.043 ± 0.0015
Se	0.161 ± 0.0823
Ag	0.052 ± 0.0823
Ti	0.050 ± 0.0002
Zn	0.041 ± 0.0031

**Table 3:** Pearson’s correlation coefficients of the antioxidant properties and minerals.

Assays	Al	As	Ba	Bi	Ca	Cd	Co	Cr	Cu	Fe	Li	Mg	Mn	Ni	Pb	Se	Ag	Ti	Zn
DPPH	−0.724	0.999 *	0.999 *	0.989	0.995	0.993	0.927	0.999 *	0.989	0.989	0.989	0.999 *	0.989	0.989	0.989	0.994	0.241	0.995	0.865
ABTS	−0.753	0.997	0.997	0.994	0.989	0.986	0.943	0.997	0.994	0.994	0.994	0.997	0.994	0.994	0.994	0.998 *	0.283	0.989	0.842
TPC	−0.700	0.972 **	0.981 **	0.983	0.998 *	0.996	0.914	0.900 **	0.983	0.983	0.983	0.990 **	0.983	0.983	0.983	0.990	0.208	0.998 *	0.882
TFC	−0.798	0.988	0.988	0.999 *	0.976	0.972	0.964	0.988	0.999 *	0.999 *	0.999 *	0.988	0.999 *	0.999 *	0.999 *	0.980 **	0.351	0.976	0.802

* Correlation is significant at the 0.05 level (two-tailed). ** Correlation is significant at the 0.01 level (two-tailed).

**Table 4:** Antioxidant activity, total phenolic content, and total flavonoid content of *Lycium chinense*.

Assays	Extracts	Control	
		BHT	Ascorbic Acid
DPPH * (IC50)	70.99 ± 7.95	34.00 ± 1.00	
ABTS * (IC50)	1772.31 ± 10.87		100.05 ± 3.50
TPC (mgGAE/g)	7.55 ± 0.22		
TFC (mgQE/g)	0.02 ± 0.001		

* The inhibitory concentration (IC_50_) value in µg/mL indicates the amount of test sample needed to inhibit or scavenge 50% of the radicals present in the reaction mixture.

**Table 5:** LC-MS/MS parameters of the phenolic compound quantitative analyses.

Phenolic Compounds	Retention Time	Q1 (*m/z*) ^a^	Q3 (*m/z*) ^b^	DP (V) ^c^	EP (V) ^d^	CEP (V) ^e^	CE (eV) ^f^	CXP (V) ^g^	Phenolic Compounds (µg/g Dry Weight of Leaf Extract)
Protocatechuic acid	8.04	152.896	108.900	−16.000	−9.000	−10.000	−22.000	−6.000	1198.70 ± 5.00
Chlorogenic acid	8.30	352.846	191.000	−21.000	−7.000	−32.000	−30.000	−10.000	192.30 ± 4.50
Rutin	9.58	609.002	299.700	−91.000	−10.500	−34.000	−52.000	−14.000	119.70 ± 2.50
*p*-Hydroxybenzoic acid	10.02	136.885	92.900	−16.000	−8.500	−12.000	−24.000	−6.000	529.70 ± 6.15
Caffeic acid	10.10	178.868	134.800	−16.000	−8.000	−14.000	−22.000	−6.000	67.60 ± 2.00
Gentisic acid	10.63	152.871	107.900	−16.000	−8.500	−6.000	−28.000	−22.000	147.90 ± 3.00
*p*-Coumaric acid	12.08	162.866	118.900	−11.000	−7.000	−14.000	−20.000	−6.000	466.90 ± 6.50
Salicylic acid	16.58	136.874	92.900	−16.000	−7.500	−12.000	−22.000	−6.000	134.30 ± 3.00

^a^ Precursor ion (Q1, *m/z*); ^b^ fragment ion (Q3, *m/z*); ^c^ DP: Declustering potential; ^d^ EP: Entrance potential; ^e^ CEP: Cell entrance potential; ^f^ CE: Collision energy; ^g^ CXP: Collision cell exit potential.

**Table 6:** Pearson’s correlation coefficients of the antioxidant properties and phenolic acids.

	ABTS	DPPH	TPC	TFC	Protocate- Chuic Acid	Chlorogenic Acid	Rutin	*p*-Hydroxy- Benzoic Acid	Caffeic Acid	Gentisic Acid	*p*-Coumaric Acid	Salicylic Acid
ABTS	1	0.999 *	0.997 *	0.997 *	0.997 *	0.949	0.990	0.999 *	0.997	0.999 *	0.999 *	0.969
DPPH	0.999 *	1	0.999 *	0.993	0.993	0.934	0.991	0.997	0.999 *	0.997 *	0.998 **	0.957

** Correlation is significant at the 0.01 level (two-tailed). * Correlation is significant at the 0.05 level (two-tailed).

**Table 7:** Pearson correlation coefficients between the phenolic compounds and cytotoxicity of the leaf extracts.

Phenolic Compounds	Cytotoxicity
Protocatechuic acid	0.957 **
Chlorogenic acid	0.998 *
Rutin	0.870
*p*-Hydroxybenzoic acid	0.872
Caffeic acid	0.919
Gentisic acid	0. 819
*p*-Coumaric acid	0.984 *
Salicylic acid	0.998 *

** Correlation is significant at the 0.01 level (two-tailed). * Correlation is significant at the 0.05 level (two-tailed).

**Table 8:** Assessment of test sample irritation potential in the HET–CAM assay.

Samples	Irritation Score	Irritation Assessment
Negative control	0	Non-irritant
NaOH (0.1 M)	18.82	Strong irritant
Leaf extracts (50 mg/mL)	0	Non-irritant

**Table 9:** Evaluation of the leaf extract minimum inhibitory concentration (MIC) against selected pathogens.

Bacterial Strains	-------MIC (ppm)-----
*B. cereus*	625
*K. pneumoniae*	>1000
*S. aureus*	625
*S. typhimurium*	>1000
*B. subtilis*	>1000
*E. coli*	155
*H. pylori*	625

**Table 10:** Antimicrobial activity of the leaf extracts based on the zone of inhibition against selected pathogens.

Bacterial Strains	Leaf Extracts	Tetracycline (10 mg)
	-----------Zone of Inhibition (cm)---------	
*B. cereus*	1.4	1.6
*K. pneumoniae*	0.5	0.8
*S. aureus*	0.9	2.0
*S. typhimurium*	0.9	1.1
*B. subtilis*	1.2	1.2
*E. coli*	1.8	1.9
*H. pylori*	1.7	1.8

## References

[1] Negi P., Jayaprakasha G., Jena B. (2003). Antioxidant and antimutagenic activities of pomegranate peel extracts. Food Chem..

[2] Zahin M., Aqil F., Ahmad I. (2010). Broad spectrum antimutagenic activity of antioxidant active fraction of Punica granatum L. peel extracts. Mutat. Res. Toxicol. Environ. Mutagen..

[3] Edward-Jones V., Rai M.K., Kon K.V. (2013). Alternative antimicrobial approaches to fighting multidrug-resistant infections. Fighting Multidrug Resistance with Herbal Extracts, Essential Oils and Their Components.

[4] Aras A., Dogru M., Bursal E. (2016). Determination of antioxidant potential of *Nepeta nuda* subsp. lydiae. Anal. Chem. Lett..

[5] Olalere O.A., Abdurahman N.H., Yunus R.B.M., Alara O.R., Ahmad M.M., Abayomi O.O., Nour A.H., Ruth A.O. (2019). Mineral element determination and phenolic compounds profiling of oleoresin extracts using an accurate mass LC-MS-QTOF and ICP-MS. J. King Saud. Univ. Sci..

[6] Sofo A., Lundegårdh B., Mårtensson A., Manfra M., Pepe G., Sommella E., De Nisco M., Tenore G.C., Campiglia P., Scopa A. (2016). Different agronomic and fertilization systems affect polyphenolic profile, antioxidant capacity and mineral composition of lettuce. Sci. Hortic..

[7] Liu X., Ardo S., Bunning M., Parry J., Zhou K., Stushnoff C., Stoniker F., Yu L., Kendall P. (2007). Total phenolic content and DPPH radical scavenging activity of lettuce (*Lactuca sativa* L.) grown in Colorado. LWT.

[8] Szymczycha-Madeja A., Welna M., Pohl P. (2012). Elemental analysis of teas and their infusions by spectrometric methods. TrAC Trends Anal. Chem..

[9] Yanai N., Shiotani S., Hagiwara S., Nabetani H., Nakajima M. (2008). Antioxidant Combination Inhibits Reactive Oxygen Species Mediated Damage. Biosci. Biotechnol. Biochem..

[10] Yokel R.A., Florence R.L. (2008). Aluminum bioavailability from tea infusion. Food Chem. Toxicol..

[11] Michalak A. (2006). Phenolic compounds and their antioxidant activity in plants growing under heavy metal stress. Pol. J. Environ. Stud..

[12] Abu-Darwish M.S., Abu-Dieyeh Z.H., Mufeed B., Al-Tawaha A.R.M., Al-Dalain S.Y.A. (2009). Trace element contents and essential oil yields from wild thyme plant (*Thymus serpyllum* L.) grown at different natural variable environments, Jordan. J. Food Agric. Environ..

[13] Nuapia Y., Chimuka L., Cukrowska E. (2018). Assessment of heavy metals in raw food samples from open markets in two African cities. Chemosphere.

[14] Kozarski M., Klaus A.S., Jakovljevic D., Todorovic N., Vunduk J., Petrovic P., Niksic M., Vrvić M., Van Griensven L.J. (2015). Antioxidants of edible mushrooms. Molecules.

[15] Velioglu Y.S., Mazza G., Gao L., Oomah B.D. (1998). Antioxidant activity and total phenolics in selected fruits, vegetables, and grain products. J. Agric. Food Chem..

[16] Safer A.M., Al-Nughamish A.J. (1999). Hepatotoxicity induced by the anti-oxidant food additive, butylated hydroxytoluene (BHT), in rats: An electron microscopical study. Histol. Histopathol..

[17] Schuenzel K.M., Harrison M.A. (2002). Microbial antagonists of foodborne pathogens on fresh, minimally processed vegetables. J. Food Prot..

[18] Sokmen A., Gulluce M., Akpulat H.A., Daferera D., Tepe B., Polissiou M., Sokmen M., Şahin F. (2004). The in vitro antimicrobial and antioxidant activities of the essential oils and methanol extracts of endemic Thymus spathulifolius. Food Control..

[19] Tepe B., Daferera D., Sökmen M., Polissiou A.M., Sökmen A. (2004). In Vitro antimicrobial and antioxidant activities of the essential oils and various extracts of Thymus eigiiM. Zohary et P.H. Davis. J. Agric. Food Chem..

[20] Paphitou N.I. (2013). Antimicrobial resistance: Action to combat the rising microbial challenges. Int. J. Antimicrob. Agents.

[21] Myers L.P., Fan R., Zheng Q., Pruett S.B. (2005). Sodium methyldithiocarbamate causes thymic atrophy by an indirect mechanism of corticosterone up-regulation. J. Immunotoxicol..

[22] Kim J.-S., Chung H.Y. (2009). GC-MS analysis of the volatile components in dried boxthorn (*Lycium chinensis*) Fruit. J. Korean Soc. Appl. Boil. Chem..

[23] Potterat O. (2009). Goji (*Lycium barbarum* and *L. chinense*): Phytochemistry, pharmacology and safety in the perspective of traditional uses and recent popularity. Planta Medica.

[24] Lin C., Chuang S., Lin J., Yang J. (1997). Evaluation of the antiinflammatory hepatoprotective and antioxidant activities of *Lycium chinense* from Taiwan. Phytomedicine.

[25] Chin Y.W., Lim S.W., Kim S.H., Shin D.Y., Suh Y.G., Kim Y.B., Kim Y.C., Kim J. (2003). Hepatoprotective pyrrole derivatives of *Lycium chinense* fruits. Bioorg. Med. Chem. Lett..

[26] Kim S.Y., Choi Y.-H., Huh H., Kim J., Kim Y.C., Lee H.S. (1997). New antihepatotoxic cerebroside from Lyciumchinense fruits. J. Nat. Prod..

[27] Ionica M.E., Nour V., Trandafir I. (2012). Polyphenols content and antioxidant capacity of goji fruits (*Lycium chinense*) as affected by the extraction solvents. South West. J. Hortic. Biol. Environ..

[28] Kim J.S. (2012). Comparison of antioxidant properties of water and ethanol extracts obtained from dried boxthorn (*Lycium chinensis*) fruit. Food Nutr. Sci..

[29] Liu S.-C., Lin J.-T., Hu C.-C., Shen B.-Y., Chen T.-Y., Chang Y.-L., Shih C.-H., Yang D.-J. (2017). Phenolic compositions and antioxidant attributes of leaves and stems from three inbred varieties of *Lycium chinense* Miller harvested at various times. Food Chem..

[30] Zhang J.-X., Guan S.-H., Feng R.-H., Wang Y., Wu Z.-Y., Zhang Y.-B., Chen X.-H., Bi K.-S., Guo D.-A. (2013). Neolignanamides, lignanamides, and other phenolic compounds from the root bark of *Lycium chinense*. J. Nat. Prod..

[31] Liu C.L., Chen Y.S., Yang J.H., Chiang B.H., Hsu C.K. (2007). Trace element water iproves the antioxidant activity of Buckwheat (Fagopyrum esculentum Moench) sprouts. J. Agric. Food Chem..

[32] Da Silva A.L.O., Barrocas P.R.G., Jacob S.D.C., Moreira J.C. (2005). Dietary intake and health effects of selected toxic elements. Braz. J. Plant Physiol..

[33] Perna A.M., Simonetti A., Intaglietta I., Sofo A., Gambacorta E. (2012). Metal content of southern Italy honey of different botanical origins and its correlation with polyphenol content and antioxidant activity. Int. J. Food Sci. Technol..

[34] Solayman M., Islam A., Paul S., Ali Y., Khalil I., Alam N., Gan S.H. (2015). Physicochemical properties, minerals, trace elements, and heavy metals in honey of different origins: A comprehensive review. Compr. Rev. Food Sci. Food Saf..

[35] Fukai T., Ushio-Fukai M. (2011). Superoxide dismutases: Role in redox signaling, vascular function, and diseases. Antioxid. Redox Signal..

[36] Bursal E. (2013). Kinetic properties of peroxidase enzyme from chard (Beta vulgaris Subspeciescicla) leaves. Int. J. Food Prop..

[37] Bingol M.N., Bursal E. (2018). LC-MS/MS analysis of phenolic compounds and in vitro antioxidant potential of stachys lavandulifolia vahl. var. brachydon boiss. Int. Lett. Nat. Sci..

[38] Nishimura F.D.C.Y., De Almeida A.C., Ratti B.A., Ueda-Nakamura T., Nakamura C.V., Ximenes V.F., Silva S.D.O. (2013). Antioxidant effects of quercetin and naringenin are associated with impaired neutrophil microbicidal activity. Evid. Based Complement. Altern. Med..

[39] Parhiz H., Roohbakhsh A., Soltani F., Rezaee R., Iranshahi M. (2014). Antioxidant and anti-inflammatory properties of the citrus flavonoids hesperidin and hesperetin: An updated review of their molecular mechanisms and experimental models. Phytother. Res..

[40] Kamalakkannan N., Prince P.S.M. (2006). Antihyperglycaemic and antioxidant effect of rutin, a polyphenolic flavonoid, in streptozotocin-induced diabetic wistar rats. Basic Clin. Pharmacol. Toxicol..

[41] Kikuzaki H., Hisamoto M., Hirose K., Akiyama K., Taniguchi H. (2002). Antioxidant properties of ferulic acid and its related compounds. J. Agric. Food Chem..

[42] Amarowicz R., Pegg R., Rahimi-Moghaddam P., Barl B., Weil J. (2004). Free-radical scavenging capacity and antioxidant activity of selected plant species from the canadian prairies. Food Chem..

[43] Balasundram N., Sundram K., Samman S. (2006). Phenolic compounds in plants and agri-industrial by-products: Antioxidant activity, occurrence, and potential uses. Food Chem..

[44] Zhang H.-Y., Sun Y.-M., Wang X.-L. (2003). Substituent effects on O—H bond dissociation enthalpies and ionization potentials of catechols: A DFT study and its implications in the rational design of phenolic antioxidants and elucidation of structure–activity relationships for flavonoid antioxidants. Chem. Eur. J..

[45] Moghaddam M., Mehdizadeh L. (2015). Variability of total phenolic, flavonoid and rosmarinic acid content among Iranian basil accessions. LWT.

[46] Neuhouser M.L. (2004). Flavonoids and cancer prevention: What is the evidence in humans?. Pharm. Biol..

[47] Mokrani A., Madani K. (2016). Effect of solvent, time and temperature on the extraction of phenolic compounds and antioxidant capacity of peach (*Prunus persica* L.) fruit. Sep. Purif. Technol..

[48] Li F., Li S., Li H.-B., Deng G.-F., Ling W.-H., Xu X.-R. (2013). Antiproliferative activities of tea and herbal infusions. Food Funct..

[49] Fontes S.T., Fernández M.R., Ogliari F.A., De Carvalho R.V., Moraes R.R., Pinto M.B., Piva E. (2012). Tetrahydrofuran as solvent in dental adhesives: Cytotoxicity and dentin bond stability. Clin. Oral Investig..

[50] Georgiev K.D., Slavov I.J., Iliev I. (2019). Synergistic growth inhibitory effects of Lycium barbarum (Goji berry) extract with doxorubicin against human breast cancer cells. J. Pharm. Pharmacol. Res..

[51] Rahal A., Kumar A., Singh V., Yadav B., Tiwari R., Chakraborty S., Dhama K. (2014). Oxidative stress, prooxidants and antioxidants: The interplay. BioMed Res. Int..

[52] Estevinho L.M., Pereira A., Moreira L.F., Dias L.G., Pereira E.L. (2008). Antioxidant and antimicrobial effects of phenolic compounds extracts of Northeast Portugal honey. Food Chem. Toxicol..

[53] Aleksic V., Knezevic P. (2014). Antimicrobial and antioxidative activity of extracts and essential oils of *Myrtus communis* L. Microbiol. Res..

[54] Llorent-Martínez E.J., Ortega-Barrales P., Panneerselvam C., Mocan A., Simirgiotis M., Ceylan R., Uysal S., Aktumsek A. (2017). Evaluation of antioxidant potential, enzyme inhibition activity and phenolic profile of *Lathyrus cicera* and *Lathyrus digitatus*: Potential sources of bioactive compounds for the food industry. Food Chem. Toxicol..

[55] Araújo K.C.F., Costa E.M.D.M., Pazini F., Valadares M.C., De Oliveira V. (2013). Bioconversion of quercetin and rutin and the cytotoxicity activities of the transformed products. Food Chem. Toxicol..

[56] Bonechi C., Donati A., Leone G., Consumi M., Lamponi S., Tamasi G., Rossi C., Magnani A. (2018). Protective effect of quercetin and rutin encapsulated liposomes on induced oxidative stress. Biophys. Chem..

[57] Tamasi G., Baratto M.C., Bonechi C., Byelyakova A., Pardini A., Donati A., Leone G., Consumi M., Lamponi S., Magnani A. (2019). Chemical characterization and antioxidant properties of products and by-products from *Olea europaea* L. Food Sci. Nutr..

[58] Danihelova M., Veverka M., Šturdík E., Jantova S. (2013). Antioxidant action and cytotoxicity on HeLa and NIH-3T3 cells of new quercetin derivatives. Interdiscip. Toxicol..

[59] Lokman N.A., Elder A.S.F., Ricciardelli C., Oehler M.K. (2012). Chick chorioallantoic membrane (CAM) assay as an in vivo model to study the effect of newly identified molecules on ovarian cancer invasion and metastasis. Int. J. Mol. Sci..

[60] Zheng W., Wang S.Y. (2001). Antioxidant activity and phenolic compounds in selected herbs. J. Agric. Food Chem..

[61] Saavedra M.J., Borges A., Dias C., Aires A., Bennett R.N., Rosa E., Simões M. (2010). Antimicrobial activity of phenolics and glucosinolate hydrolysis products and their synergy with streptomycin against pathogenic bacteria. Med. Chem..

[62] Hirai I., Okuno M., Katsuma R., Arita N., Tachibana M., Yamamoto Y. (2010). Characterisation of anti-*Staphylococcus aureus* activity of quercetin. Int. J. Food Sci. Technol..

[63] Cueva C., Moreno-Arribas M.V., Martin-Álvarez P.J., Bills G.F., Vicente M.F., Basilio A., Rivas C.L., Requena T., Rodríguez J.M., Bartolomé B. (2010). Antimicrobial activity of phenolic acids against commensal, probiotic and pathogenic bacteria. Res. Microbiol..

[64] Liang Y., Xu Q., Xie H., Zhou Y., Wei X. (2010). Chemical constituents from mango seed kernels and their antimicrobial activity. J. Trop. Subtrop. Bot..

[65] Lou Z., Wang H., Zhu S., Ma C., Wang Z. (2011). Antibacterial activity and mechanism of action of chlorogenic acid. J. Food Sci..

[66] Singleton V.L., Rossi J.A. (1965). Colorimetry of total phenolics with phosphomolybdic-phosphotungstic acid reagents. Am. J. Enol. Vitic..

[67] Moreno M.I., Isla M.I., Sampietro A.R., Vattuone M.A. (2000). Comparison of the free radical-scavenging activity of propolis from several regions of Argentina. J. Ethnopharmacol..

[68] Chung I.-M., Chelliah R., Oh D.-H., Kim S.-H., Yu C.Y., Ghimire B.K. (2019). Tupistra nutans wall. root extract, rich in phenolics, inhibits microbial growth and α-glucosidase activity, while demonstrating strong antioxidant potential. Braz. J. Bot..

[69] Xiong Q., Kadota S., Tani T., Namba T. (1996). Antioxidative effects of phenylethanoids from cistanche deserticola. Boil. Pharm. Bull..

[70] Thaipong K., Boonprakob U., Crosby K., Cisneros-Zevallos L., Byrne D.H. (2006). Comparison of ABTS, DPPH, FRAP, and ORAC assays for estimating antioxidant activity from guava fruit extracts. J. Food Compos. Anal..

[71] Sandra F., Sudiono J., Trisfilha P., Pratiwi D. (2017). Cytotoxicity of *Alpinia galanga* rhizome crude extract on NIH-3T3 cells. Indones. Biomed. J..

[72] Rios J., Recio M. (2005). Medicinal plants and antimicrobial activity. J. Ethnopharmacol..

[73] Kobayashi M., Kakizono T., Nagai S. (1993). Enhanced carotenoid biosynthesis by oxidative stress in acetate-induced cyst cells of a green unicellular alga, *Haematococcus pluvialis*. Appl. Environ. Microbiol..

